# UNDERSTANDING SPENDDOWN TO MEDICAID IN ASSISTED LIVING: SURVEY RESULTS FROM PARTICIPANTS/REPRESENTATIVES IN MINNESOTA

**DOI:** 10.1093/geroni/igac059.100

**Published:** 2022-12-20

**Authors:** Sharon Baggett, Ellen Burton, Justin Blackburn

**Affiliations:** University of Indianapolis, Indianapolis, Indiana, United States; University of Indianapolis Center for Aging & Community, Indianapolis, Indiana, United States; IU Richard M. Fairbanks School of Public Health at IUPUI, Indianapolis, Indiana, United States

## Abstract

The state of Minnesota has seen substantial growth in the use of Medicaid in Assisted Living and wanted to better understand how applicants spend down to Medicaid in order to identify potential upstream interventions to prolong the spend down period. As part of a mixed methods research effort, 231 new Medicaid participants or their proxies, 167 in AL and 64 at home, completed a telephone survey on their services use prior to Medicaid application, prior planning or thinking of how to pay for services once personal resources were exhausted, and experience of spenddown once in AL. Reaching these eligible participants required a unique, multi-step outreach process; given the abilities of participants to respond, the majority of surveys were completed by proxies. Among those in AL, 40% reported planning in advance about how to pay for services once needed; 58% knew of government programs to assist and 80% reported their plan included applying to the programs at some time. Thirteen percent (13%) of those in AL reported getting help from the Medicaid waiver at the time of move in; another 25% said they paid for themselves for less than one year before getting state support. More than one-third (34%) who paid for their services for some period of time after move in said the period was shorter than expected; another 41% said about as expected, and for 25% the period was longer than expected. Open-ended comments indicate a move to memory care was a key factor in spend down to Medicaid.

